# Lignans

**DOI:** 10.3390/molecules24071424

**Published:** 2019-04-11

**Authors:** David Barker

**Affiliations:** School of Chemical Sciences, University of Auckland, Private Bag, Auckland 92019, New Zealand; d.barker@auckland.ac.nz

The 13 research articles/communications, six reviews, and one perspective that comprise this Special Issue on Lignans, highlight the most recent research and investigations into this diverse and important class of bioactive natural products. 

Lignans are traditionally defined as a class of secondary metabolites that are derived from the oxidative dimerization of two or more phenylpropanoid units. Despite their common biosynthetic origins, they boast a vast structural diversity. It is also well-established that this class of compounds exhibit a range of potent biological activities. Owing to these factors, lignans have proven to be a challenging and desirable synthetic target that has instigated the development of a number of different synthetic methods, advancing our collective knowledge towards the synthesis of complex and unique structures. 

New lignans are constantly being found and this Special Issue details some of the most recently discovered novel lignans—Liu et al. isolated three new dibenzocyclooctadiene lignans, heilaohulignans A–C from Heilaohu, the roots of Kadsura coccinea, which have a long history of use in Tujia ethnomedicine for the treatment of rheumatoid arthritis and gastroenteric disorders [1]. Heilaohulignan C, in particular, demonstrated cytotoxic activity in a number of human cancer cell lines. Two new lignan glycosides have also been found in the aerial portion of Lespedeza cuneata (Fabaceae), known as Chinese bushclover, a plant that has been used in traditional medicine for the treatment of diseases including diabetes, hematuria, and insomnia [2]. These newly-discovered compounds were tested for their biological activities against human breast cancer cell lines, showing some cytotoxic activity. A review detailing over 270 lignans isolated from Lauraceae, a valuable source of lignans and neolignans is also presented, compiled by Li et al. [3]. Furthermore, Mexican Bursera plants have been used in traditional medicine for treating various pathophysiological disorders and are a rich source of lignans. An Italian research group have summarized the biological activities of lignans isolated from selected Mexican Bursera plants in their review [4].

A subclass of lignans, norlignans lack a carbon present in the parent lignan structure, with 9-norlignans lacking a terminal carbon (C-9). An overview of the occurrence and biological activity of all the 9-norlignans reported to date are given in the article by Eklund and Raitanen, which also reports the semisynthetic preparation of a number of 9-norlignans using the natural lignan hydroxymatairesinol, obtained from spruce knots, as the starting material [5]. 

As stated above, owing to their potent biological activities, lignans are a popular synthetic target. A summary of the advances in lignan natural product synthesis over the last decade is outlined in the review by Fang and Hu [6]. 

Davidson et al. have presented their work on their novel, efficient, convergent, and modular synthesis of the well-known dibenzyl butyrolactone lignans through the use of the acyl-Claisen rearrangement to stereoselectively prepare a key intermediate [7]. Not only were the natural products able to be obtained, but the reported synthetic route also enabled the modification of these lignans to give rise to 5-hydroxymethyl derivatives, which were then shown to have an excellent cytotoxic profile which resulted in programmed cell death of Jurkat T-leukemia cells with less than 2% of the incubated cells entering a necrotic cell death pathway.

Advances in the synthesis of aryldihydronaphthalene and arylnapthalene lignans are also detailed in this Special Issue through the concise synthesis of (+)-β- and γ-apopicropodophyllins and dehydrodesoxypodophyllotoxin [8]. This was achieved using the key reaction involving regiocontrolled oxidations of stereodivergent aryltetralin lactones, which were easily accessed from a nickel-catalyzed reductive cascade approach. 

As stated, lignans are formed from the oxidative dimerization of two or more phenyl propanoid units. However, numerous oxidative transformations of lignans themselves have been reported in the literature. Runeberg et al. provide an overview on the current findings in this field, focusing on transformations targeting a specific structure, reaction, or an interconversion of the lignan skeleton [9]. 

The extensive analysis of the potent biological activities of lignans remains a popular avenue of investigation. Antunez-Mojica et al. used a zebrafish embryo model to guide the chromatographic fractionation of antimitotic secondary metabolites, ultimately leading to the isolation of several podophyllotoxin-type lignans from the steam bark of *Bursera fagaroides* [10]. Subsequent to their isolation, the biological effects on mitosis, cell migration, and microtubule cytoskeleton remodeling of the isolated lignans were then further evaluated in zebrafish embryos through various methods. Ultimately, it was demonstrated that the zebrafish model can be a fast and inexpensive in vivo model to identify antimitotic natural products through bioassay-guided fractionation.

Pereira Rocha et al. combined the in silico prediction of biological activities of lignans from *Diphylleia cymosa* and *Podophyllum hexandrum* with in vitro bioassays testing the antibacterial, anticholinesterasic, antioxidant, and cytotoxic activities of these lignans [11]. In this study, the in silico approach was validated and several ethnopharmacological uses and known biological activities of lignans were confirmed, whilst it was shown that others should be investigated for new drugs with potential clinical use. 

To explore the differences in lignan composition profiles between various parts and genders of *Schisandra rubriflora* and *Schisandra chinesis* (wuweizi), Szopa et al. used UHPLC-MS/MS [12]. Additionally, the anti-inflammatory activity of plant extracts and individual lignans was tested in vitro for the inhibition of 15-lipooxygenase (15-LOX), phospholipases A2 (sPLA2), cyclooxygenase 1 and 2 (COX-1; COX-2) enzyme activities. The results of anti-inflammatory assays revealed higher activity of *S. rubriflora* extracts, while individual lignans showed significant inhibitory activity against 15-LOX, COX-1 and COX-2 enzymes. Closely related, Chen et al. evaluated the quality and effect of cultivated and wild growing methods on the lignan composition of *Schisandra chinesis* through the use of UFLC-QTRAP-MS/MS in combination with multivariate statistical analysis, demonstrating that the composition differs between plants grown in these conditions and the quality of cultivated wuweizi was not as good as wild wuweizi [13].

While lignans have been shown to exhibit extensive potent biological activities, other factors need to be considered for them to be potential drugs. The physicochemical properties of various lignans subclasses were analyzed by Dr Lisa Pilkington to assess their Absorption, Distribution, Metabolism, Excretion and Toxicity (ADMET) profiles and establish if these compounds are lead-like/drug-like and thus have potential to be or act as leads in the development of future therapeutics [14]. Overall, she established that lignans show a particularly high level of drug-likeness, an observation that, coupled with their potent biological activities, demands future pursuit into their potential for use as therapeutics.

Traditionally, health benefits attributed to lignans have included a lowered risk of heart disease, menopausal symptoms, osteoporosis, and breast cancer. Rodriguez-Garcia et al. present a review that focuses on the potential health benefits attributable to the consumption of different diets containing naturally lignan-rich foods [15]. Current evidence endorses lignans as human health-promoting molecules and, therefore, dietary intake of lignan-rich foods could be a useful way to bolster the prevention of chronic illness, such as certain types of cancers and cardiovascular disease.

Lignan composition profiles of flaxseed, the richest grain source of lignans, was also studied, assessing the relative impact of genetic and geographic parameters on the phytochemical yield and composition [16]. It was found that cultivar is more influential than geographic parameters on the flaxseed phytochemical accumulation yield and composition. In addition, the corresponding antioxidant activity of these flaxseed extracts was evaluated using both in vitro, and in vivo methods, which confirmed that flaxseed extracts are an effective protector against oxidative stress and that secoisolariciresinol diglucoside, caffeic acid glucoside, and *p*-coumaric acid glucoside are the main contributors to the antioxidant capacity. A review of the use and effect of flaxseed as a food source for dairy cows has also been presented [17], covering the gastrointestinal tract metabolism of lignans in humans and animals. The review also provided an in-depth assessment of research towards the impacts of flaxseed products on milk enterolactone concentration and animal health, and the pharmacokinetics of enterolactone consumed through milk, which may have implications to both ruminants and humans’ health.

With the rise in exploration of dietary lignans and their various effects, exemplified by the aforementioned studies, the study by Durazzo et al. provides assessment and analysis of the development and management of databases on dietary lignans, which includes a description of the occurrence of lignans in food groups, the initial construction of the first lignan databases, and their inclusion in harmonized databases at national and/or European level [18]. 

In addition to work into their notable biological activities, there has been a recent increase in investigations exploring lignans in other roles. This includes gaining insight into the effects of barrel-aging on spirits, whereby lignans present in the wooden barrels are released into the aging spirit. To evaluate the impact of lignans in spirits, screening of a number of lignans was set up and served to validate their presence in the spirit and release by oak wood during aging [19]. The most abundant, and also the bitterest, lignan, (±)-lyoniresinol was detected and quantified in a large number of samples to be above the gustatory threshold, suggesting its effect of increased bitterness in spirit taste. Related to this, the molecular dynamics on wood-derived lignans were analyzed by intramolecular network theory by Sandberg et al. [20]. These wood-derived lignan-based ligands called LIGNOLs were studied, where it was found in the hydration studies that tetramethyl 1,4-diol is the LIGNOL which was most likely to form hydrogen bonds to TIP4P solvent.

In summary, it can be seen in this Special Issue that research in natural lignans and lignin-derived compounds continues to be a fruitful area of research. Scientists working across a large number of disciplines continue to be attracted to work on lignans due to their relatively high natural abundance, coupled with their highly potent and diverse range of biological activities. 
